# Resilience as a concept for understanding family caregiving of adults with Chronic Obstructive Pulmonary Disease (COPD): an integrative review

**DOI:** 10.1002/nop2.63

**Published:** 2016-10-11

**Authors:** Francesca Rosa, Annamaria Bagnasco, Giuseppe Aleo, Sally Kendall, Loredana Sasso

**Affiliations:** ^1^ Department of Health SciencesUniversity of GenoaGenoaItaly; ^2^ Centre for Research in Primary and Community Care (CRIPACC)University of HertfordshireHatfieldUK

**Keywords:** caregiver (or family caregiver), chronic disease, Chronic Obstructive Pulmonary Disease, coping, Health Assets, integrative review, literature review, quality of life, resilience

## Abstract

**Aims:**

This paper was a report of the synthesis of evidence on examining the origins and definitions of the concept of resilience, investigating its application in chronic illness management and exploring its utility as a means of understanding family caregiving of adults with Chronic Obstructive Pulmonary Disease.

**Background:**

Resilience is a concept that is becoming relevant to understanding how individuals and families live with illness, especially long‐term conditions. Caregivers of adults with Chronic Obstructive Pulmonary Disease must be able to respond to exacerbations of the condition and may themselves experience cognitive imbalances. Yet, resilience as a way of understanding family caregiving of adults with COPD is little explored.

**Design:**

Literature review – integrative review.

**Data sources:**

CINAHL, PubMed, Google Scholar and EBSCO were searched between 1989–2015.

**Review methods:**

The principles of rapid evidence assessment were followed.

**Results:**

We identified 376 relevant papers: 20 papers reported the presence of the concept of resilience in family caregivers of chronic diseases patients but only 12 papers reported the presence of the concept of resilience in caregivers of Chronic Obstructive Pulmonary Disease patients and have been included in the synthesis. The term resilience in Chronic Obstructive Pulmonary Disease caregiving is most often understood using a deficit model of health.

## 1 Introduction

Chronic Obstructive Pulmonary Disease (COPD) is a lung disease characterized by chronic obstruction of lung airflow that interferes with normal breathing and is not fully reversible (WHO http://www.who.int/respiratory/copd/definition/en/). Damage to lungs from COPD cannot be reversed, but treatment can help to control symptoms and minimize further damage (Mayo Clinic http://www.mayoclinic.org/diseases-conditions/copd/basics/definition/con-20032017). COPD is among the leading 10 causes of death worldwide (World Health Organization, 2002).

An exacerbation refers to sustained worsening of the patient's symptoms from their usual stable state, which is beyond normal day‐to‐day variations and has an acute onset. (National Clinical Guideline Centre, 2010). Chronic Obstructive Pulmonary Disease (COPD) is now a major public health concern. More than 3 million people died of COPD in 2012, which is equal to 6% of all deaths globally that year (World Health Organization, 2005)

COPD is common in later life: an estimated 3 million people have COPD in the UK. Although for approximately 2 million of this group their COPD remains undiagnosed (Healthcare Commission, 2006) 2535 people died from mesothelioma in 2012, and thousands more from other occupational cancers and diseases such as COPD (Health and Safety Executive, 2013). In Italy, 2.6 million people are diagnosed with COPD and there are 18,000 reported mortalities every year from this condition (Del Negro & Rossi, 2003).

Hospital‐at‐home and assisted‐discharge strategies are safe and effective, and current Department of Health guidance recommends such approaches should be preferred as an alternative way of caring for patients with exacerbations of COPD. They otherwise need to be admitted to hospital (National Clinical Guideline Centre, 2010). However, this often leads to increased care responsibilities for families, who are required to carry greater care burdens for longer periods of time (Grant, Cavanagh, & Yorke, 2012). Approximately 43.5 million US adults provide an average of 19 hours of unpaid informal care per week for someone aged 50 and older (National Alliance for Caregiving, AARP, 2013; Sautter, Tulsky, & Johnson, 2014). Family caregiving refers to unpaid relatives or friends of a disabled individual who help that individual with his or her activities of daily living. The words may be prefixed with ‘family’ ‘spousal’, ‘child’, ‘parent’, ‘young’ or ‘adult’ (Kumar, Matreja, Gupta, Singh, & Garg, 2012).

## 2 Background

Caregivers of patients with COPD (Chronic Obstructive Pulmonary Disease) must be able to respond to exacerbations and may experience cognitive, emotional, social imbalances, depression, anxiety and stress (Pearlin, Mullan, Semple, & Skaff, 1990; Simpson, Young, & Donahue, 2010; Zarit, Tood, & Zarit, 1986). Government policies should help informal caregivers to receive practical support so that they may continue to care for their beloved ones in the long term without damaging their own health and well‐being, because informal caregivers provide a service that would significantly weight on health and social services in terms of costs (Pinto, Holanda, Medeiros, Mota, & Pereira, 2007).

Traditionally, nursing care has focused primarily on the negative impact that caregiving has on caregivers and to solve these problems on behalf of the caregiver (Day & Anderson, 2011). However, the problem‐oriented approach to nursing care is no longer sufficient, and the growing focus on self‐management of illness in chronic diseases, on patient‐centred care, and patient empowerment is the proof of this (Rotegård, Moore, Fagermoen, & Ruland, 2010). According to the Health Assets Model, helping patients and caregivers achieve and maintain their health and wellness is essential. “An assets approach to health and development embraces a ‘salutogenic’ notion of health creation and in doing so encourages the full participation of local communities in the health development process” (Antonovsky, 1979; Morgan, Davies, & Ziglio, 2010; Morgan & Ziglio, 2007). Resilience is a concept that is becoming relevant for understanding how individuals and families live with illness, especially long‐term conditions. Yet, resilience as a way of understanding family caregiving of adults with COPD is little explored. The present paper will present an in‐depth analysis of resilience as a concept for understanding family caregiving of adults with COPD to provide a clearer conceptual basis for research in this field. Greater clarity about the concept may also help nurses who work with caregivers to provide more effective support. COPD management should also focus on symptom management and home care throughout the course of the disease, not only on optimal drug therapy (Nakken et al., 2015).

Initially, a concept analysis was considered as a valuable and rigorous method to clarify the meaning and nature of resilience in this group as it can aid precise communication, critical thinking and the advancement of the knowledge base of nursing (Cahill, 1996). However, an initial review of the literature of family caregiving of adults with COPD revealed that the term ‘resilience? is not used either by researchers or practitioners working in this field. It was decided, therefore, to adopt a broader perspective and conduct a review of the literature to examine the origins and definitions of the concept, investigate its application in other areas of chronic illness management, and explore its utility as a means of understanding family caregiving in adults with COPD.

### 2.1 Origins and definitions of ‘resilience’

Initially, a search for definitions of the term ‘resilience’ was conducted followed by a literature review on concept analyses defining resilience and related concepts in the period 1980–2015. Before 1990, most of the research on resilience was limited to children and adolescents; after 1990 more studies were conducted in adults (Tusaie & Dyer, 2004). Similarly, prior to 20 years ago, resilience was studied predominantly as a trait that people either did or did not have (Earvolino‐Ramirez, 2007), whereas more recently it has been seen as a quality that can be developed (Hicks & Conner, 2013).

The term ‘resilience’ derives from the Latin word ‘resilient’ which means ‘act of rebounding’ present participle of ‘resilire’ ‘to rebound, recoil,’ (Online etymology dictionary). Oxford Dictionaries (online) defines resilience as ‘The capacity to recover quickly from difficulties; toughness’. According to Merriam‐Webster dictionary, resilience is ‘an ability to recover from or adjust easily to misfortune or change’ while WordNet.com describes the term as ‘the occurrence of rebounding or springing back’. The ability to recover from an altered state or a sense of recovery and rebounding is common to all definitions.

The concept of resilience originated from early psychiatric literature (Earvolino‐Ramirez, 2007) and has been applied to different biological and human activities or sciences, like ecology, engineering and business. Modern resilience studies originated among psychologists and psychiatrists during the 1980s and 1990s, who were concerned to challenge a deficit, pathological, model of stress and coping and understand how individual, family and societal strengths contribute to mental well‐being. Health researchers also increasingly recognized that strengths and capacities are important resources for promoting good health (Lin, Rong, & Lee, 2013).

Garcia‐Dia, DiNapoli, Garcia‐Ona, Jakubowski, and O’Flaherty (2013) defines resilience as ‘a dynamic process that can be influenced by the environment, external factors and/or the individual and the outcome’. Family and community are listed as external factors that can influence the development of resilience. Four defining attributes have been identified for the concept of Resilience by Garcia‐Dia et al. (2013): ‘Rebounding’, described as the ability to bounce back after facing a life altering event; ‘Determination’, described as a firm or fixed intention to achieve a desired end; ‘Social Support and Self‐efficacy’, described as the belief in one's own ability to achieve a goal or overcome an event.

Resilient caregivers are proactive towards maintaining harmonious relationships with health care professionals, aggregate information and resources, and developing social networks (Lin et al., 2013). Caregivers, who face the challenges of taking care of their beloved ones, express their feelings relying on their social support networks to help them deal with their life situations (Garcia‐Dia et al., 2013).

Sometimes, there can be unexpected pathways to resilience (Bonanno, 2004). Bonanno defines resilience as ‘the ability of adults in otherwise normal circumstances, who were exposed to an isolated and potentially highly disruptive event, to maintain relatively stable and healthy levels of psychological and physical functioning, and the capacity for generative experiences and positive emotions’.

Hicks and Conner (2013) analysed the concept of ‘resilient ageing’ and defined the antecedents, attributes and consequences of resilient ageing: antecedents to resilient ageing were found to be adversity and protective factors, while the core attributes include coping, hardiness and self‐concept. Cowan, Cowan, and Schultz (1996) defined family resilience as ‘an adaptive capacity or strength, postulating it as an adaptive capacity for balance in a family when confronting crises, and as a potential power of the family that activates flexibility, problem solving, and resource mobilization within the family’.

Resilience in the workplace has been progressively studied in the nursing profession (Benner & Wrubel, 1989). In addition, resilience is one of the individual and collaborative factors that are known to mediate stress in cancer settings (Zander, Hutton, & King, 2010).

## 3 The review

### 3.1 Aims

The aim of this Integrative Review was to analyse peer‐reviewed studies focusing on resilience in family caregivers of chronic disease patients, synthesize the findings and explore its utility as a means of understanding family caregiving of adults with COPD. The review considered the following questions:

- Is the concept of resilience used in the literature focused on family caregivers of patients with chronic conditions?
- What are the terms used when describing caregiving of family caregivers of chronic conditions?
- Which type of model is used in these studies? A Deficit or an Assets Model of Health?
- Is the concept of resilience applicable to caregivers of COPD patients?

To manage and break down the research question, we used the PEO format (Bettany‐Saltikov, 2012) to identify the key concepts in our research question, and define the inclusion and exclusion criteria (Bianchi et al., 2016).

**Table:** 

P	Population	Caregivers of patients with chronic conditions.
E	Exposure	Caregiving of patients with chronic conditions.
O	Outcomes	1) Resilience in caregivers of patients with chronic conditions.2) Resilience is a concept applicable to family caregivers of COPD patients.

### 3.2 Design

This is an integrative review (Whittemore & Knafl, 2005), and a Rapid Evidence Assessment (REA 2011). An updated integrative review method has the potential to allow for diverse primary research methods to become a greater part of evidence‐based practice initiatives (Earle, 2011) (Whittemore & Knafl, 2005). A Rapid Evidence Assessment is a rigorous method that gathers and reviews evidence in a streamlined systematic way (Blank et al., 2012).

### 3.3 Search method

As well as exploring the concept of resilience, it was important also to examine the literature that used an alternative perspective to understand family caregiving in chronic illness. A literature review (1989–2015) was conducted using the following keywords: resilience, caregiver (or family caregiver), Chronic Obstructive Pulmonary Disease, and chronic disease. The databases used in the search were: CINAHL, PubMed, Google Scholar, and EBSCO.

**Figure 1:**
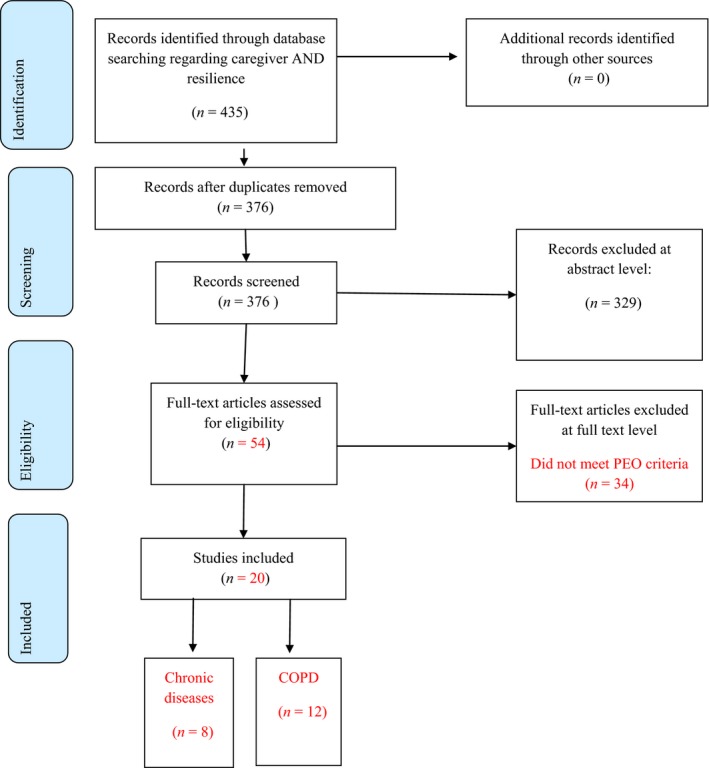
Flow diagram literature review resilience in chronic diseases

First, we selected studies based on titles, keywords and abstracts. Then, we analysed the full‐text articles. After collecting the evidence, the eligibility criteria were applied to the results and all identified references were screened independently by reviewers using an assessment according to the Preferred Reporting Items for Systematic Reviews and Meta‐Analyses (PRISMA) statement (Moher, Liberati, Tetzlaff, & Altman, 2009) and PRISMA check list (Figure 1).

Empirical and Theoretical articles were included if they met the following inclusion criteria:

- Research articles related to resilience and caregivers or family caregivers in chronic diseases;
- Articles focusing on resilience and caregivers of adults with COPD, resilience in caregivers in chronic diseases;
- Written in English;
- Published in a peer‐reviewed journal;
- Involving humans

These inclusion criteria were used to obtain the most relevant papers, as literature relating to chronic illness is extremely extensive.

### 3.4 Search outcomes

Our searches generated 435 potentially relevant papers. After duplicates were removed, the remaining records were 376. Through abstract reading and analysing, 329 articles were excluded because they did not meet the inclusion criteria of the study. Full paper appraisal (54) resulted in the exclusion of 34 articles, because they did not meet PEO criteria. Relevant papers (20) were analysed. Of these, 12 papers focused on COPD patients and caregivers, whereas eight focused on other chronic diseases (Figure 1).

### 3.5 Quality appraisal

The papers included in this review had quantitative, qualitative, and mixed‐method study designs. The critical appraisal of the papers retrieved was conducted in three stages: assessment of relevance, data extraction and scoring for methodological rigour (Hawker, Payne, Kerr, Hardey, & Powell, 2002) (Table 1).

**Table 1:** Describes the studies included the present review focusing on resilience in caregivers of patients affected by chronic diseases. It shows if the article uses a deficit or an assets model, and the methodological rigour score

Author	Title	Journal	Population	Design	Method	Results	Impact of the disease on caregivers/protective factors	Deficit/assetsmodel	Quality ApprovedTotal score Max. score = 36
Cain (2000)	Caregiver attributes as correlates of burden in family caregivers coping with chronic obstructive pulmonary disease	Journal of Family Nursing	Family Care givers in COPD patients	Descriptive design was employed in this secondary data analysis.	Data from a convenience sample of 138 family caregivers of 138 patients diagnosed with COPD were analysed to answer the research questions.	Caregivers in this sample experienced stress, operationalized as caregiver burden.	Resources, opportunities, and choices may determine whether caregivers experience subjective burden.	Deficit model	30
Caress, Luker, Chalmers, and Salmon (2009)	Promoting the health of people with chronic obstructive pulmonary disease: patients’ and carers’ views.	Journal of Clinical Nursing	Patients and carers in chronic obstructive pulmonary disease	Exploratory, descriptive design was employed	Semi‐structured, audio‐recorded interviews were conducted with 14 patients and 12 family caregivers.	The three main themes were ‘health promotion: what's that?’, ‘community resources for health promotion’ and ‘it wasn't just the smoking’.	Carers often felt at a loss as to how best to help and support the patient with COPD and many would welcome educational or other interventions to facilitate them in doing so.	Deficit model	34
Dias (2015)	Resilience of caregivers of people with dementia: a systematic review of biological and psychosocial determinants.	Trends Psychiatry Psychotherapy	Caregivers of people with dementia	Review	This study systematically reviewed the definitions, methodological approaches and determinant models associated with resilience among caregivers of people with dementia.	Resilience has been defined as positive adaptation to face adversity, flexibility, psychological well‐being, strength, healthy life, burden, social network and satisfaction with social support. No consensus was found about the definition of resilience associated with dementia.	Higher levels of resilience were associated with lower depression rates and greater physical health. Lower burden, stress, neuroticism and perceived control were the main psychological factors associated with resilience. Social support was a moderating factor of resilience, and different types of support seemed to relieve the physical and mental overload caused by stress.	Deficit model	34
Gabriel et al. (2014)	Day‐to‐day living with severe chronic obstructive pulmonary disease: towards a family‐based approach to the illness impacts.	Psychology Health	Patients and family members in COPD.	An exploratory qualitative study, with a cross‐sectional design,	A structured questionnaire was used to collect socio‐demographic data from patients and family member	Given the demands of the disease, family members felt that the patient required more attention and care, leading to restrictions in their social life (n = 18) some spouses also revealed that they felt limited to home due to patient's dependence of oxygen therapy	The overall findings illustrate the complex interaction between the experience of living with COPD and communication patterns, emotional states, social support and social role within the family.	Deficit Model	32
Grant et al. (2012)	The impact of caring for those with chronic obstructive pulmonary disease (COPD) on carers’ psychological well‐being: A narrative review	International Journal of Nursing Studies	Carers of COPD patients	Review	Studies if reported the carers perspective of caregiving – studies that focused mostly on the person with chronic obstructive pulmonary disease were included only if the carers perspective of the caregiver role could be extracted.	Many factors are related to caregiver psychological distress, but it is not possible to gauge the prevalence of this at present.	Further studies are needed to clarify the prevalence of chronic obstructive pulmonary disease caregivers’ psychological comorbidity and disease specific factors that predict poorer carer health outcomes.	Deficit model	34
Harmell et al. (2011)	A review of the psychobiology of dementia caregiving: a focus on resilience factors.	Curr Psychiatry Rep	Informal dementia caregivers	Review	A PubMed search using the search terms, *“coping and biomarker” and “resilience and biomarker”* for the years of 2000–2008 yielded a total of 160 articles on the relations between coping/resilience factors and biomarker outcomes.	We highlight 11 studies that examined the relationship of one of three broad resilience domains (personal mastery, self‐efficacy and coping style) to caregiver health outcomes.	Higher levels of personal mastery and self‐efficacy, and increased use of positive coping strategies appear to have a protective effect on various health outcomes in dementia caregivers.	Asset Model	33
Hynes et al. (2010)	Informal caregiving in advanced chronic obstructive pulmonary disease: lay knowledge and experience	Journal of Clinical Nursing	Informal caregiving in advanced chronic obstructive pulmonary disease	A qualitative exploratory approach	Semi‐structured interviews with 11 family caregivers of people with advanced chronic obstructive pulmonary disease	Six core themes emerged including ‘then and now’ reflecting caregivers’ sense of loss and enmeshment with the illness experience and burden.	The caregivers’ experience of illness burden included symptom, cultural and lifeworld meanings.	Deficit model	34
El Masry et al. (2013)	Psychosocial experiences and needs of Australian caregivers of people with stroke: prognosis messages, caregiver resilience, and relationships.	Top Stroke Rehabil	Twenty Australian informal caregivers and 10 stroke survivors	Qualitative	Individual semi‐structured qualitative interviews	The five interrelated topics most discussed were changes in relationships and support services, including being told to expect a poor outcome; caregiver attributes and coping strategies; stroke survivor limitations; external employment and financial stressors; and unexpected positive changes in relationships and priorities.	Overall, data indicate that stroke caregivers underwent a series of psychological, emotional, interpersonal, social, health, and occupational changes as a result of undertaking this role. Caregivers exhibited several different cognitive and behavioural coping strategies for managing their situation.	Assets model Some caregivers focused on realistic (rational/factual) and even positive aspects of their situation rather than filtering negative information.	33
Lee et al. (2004)	Concept development of family resilience: a study of Korean families with a chronically ill child.	Journal of Clinical Nursing	Korean families with a chronically ill child	Concept analysis	Twenty‐one conceptual attributes of family resilience emerging from this study were differentiated into four dimensions:1. Intrinsic family characteristics.2. Family member orientation related to family characteristics.3. Responsiveness to stress.4.External orientation.	Family resilience is “enduring force that leads a family to change its functioning dynamics in order to solve problems encountered”Their stories told of a continuing process of major and minor biographical life changes as care recipients’ illness progressed. A common response to these ongoing changes was to apply a day at a time framework to life.	Findings reflect substantial caregiver vulnerability in terms of an imbalance between burden and coping capacity.Subthemes were: loss of intimate relationship/identity, disease‐related demands and coping‐related factors.	Deficit model/assets modelCaregivers described their experience as a series of ‘ups‐and‐downs.’	34
Lin et al. (2013)	Resilience among caregivers of children with chronic conditions: a concept analysis	Journal of Multidisciplinary Healthcare	Caregivers of children with chronic conditions	The study includes a literature review of conceptual definitions of caregiver resilience in caring for children with chronic conditions.	Walker and Avant's methodology guided the analysis.	This concept analysis provides guidance for clinicians working with caregivers of chronically ill children. In the allied health literature, the concept of resilience is measured indirectly, most often by measuring the family	We found caregivers tended to focus on the positive, and the caregivers’ belief was that child with a chronic condition is a special favour for them. Resilient caregivers are proactive towards gathering information and resources, maintaining cooperative relationships with health care professionals, and developing social networks.	Assets model	34
Marques et al. (2015)	Family‐Based Psychosocial Support and Education as Part of Pulmonary Rehabilitation in COPD: A Randomized Controlled Trial	Chest	Family dyads (patient and family member in COPD)	A Randomized Controlled Trial	Family dyads (patient and family member) were randomly assigned to family‐based (experimental) or conventional (control) PR.	The main findings indicate that integrating the family member in PR contributed to improve the coping strategies of the family to manage the disease, with further improvement in family members’ sexual functioning and psychological distress.	Family‐based pulmonary rehabilitation benefits the family by improving the coping strategies and the psychosocial adjustment to illness. To contribute to integrated care towards managing COPD, PR programs should consider actively involving the family system within the care delivery.	Deficit Model	33
Meier et al. (2011)	Dyadic coping, quality of life, and psychological distress among chronic obstructive pulmonary disease patients and their partners.	International Journal Chronic Obstructive Pulmonary Disease	43 couples	Questionnaire	Mailed questionnaires on anxiety and depression (Hospital Anxiety and Depression Scale), quality of life (World Health Organization Quality of Life Questionnaire‐BREF), and dyadic coping (Dyadic Coping Inventory).	The higher the patient perceived the imbalance in delegated dyadic coping, the lower the couple's quality of life. More negative and less positive dyadic coping were associated with lower quality of life and higher psychological distress.	Psychotherapeutic interventions to improve dyadic coping may lead to better quality of life and less psychological distress among COPD patients and their partners.	Deficit Model	33
Nakken et al. (2015)	Informal caregivers of patients with COPD: Home Sweet Home?	European respiratory review	Patient and caregiver COPD	Literature review	This article reviews the current knowledge about these informal caregivers of patients with COPD, the impact of COPD on their lives and their perception of the patient's health status.	To conclude, patients with COPD and their informal caregivers are confronted daily with multiple limitations due to COPD. Therefore, COPD management should not only focus on the optimal drug therapy, but also on its home management throughout the whole disease trajectory. Informal caregivers play an important role, but the process of informal caregiving is complex.	Exploring the interaction between patients and informal caregivers and paying attention to the needs of informal caregivers should be part of research and in turn, of regular clinical care for patients with COPD.	Assets Model	31
Pinto et al. (2007)	Burden of caregiving for patients with chronic obstructive pulmonary disease	Respiratory Medicine	Caregivers	A cross‐sectional study was carried out with 42 COPD patients and their primary caregivers.	Patients were assessed with the medical outcome survey short form (SF‐36), the physical and mental component summary (PCS and MCS), Saint George's respiratory questionnaire (SGRQ), 6‐min walking test, and spirometric and blood gas measurements. Caregivers were assessed using the medical outcome survey short form (SF‐36), the physical and mental component summary (PCS and MCS), the 5‐point	The two most important predictors of COPD burden are the relationship between caregivers and patients and caregiver MCS scores.About 50% of our caregivers reported comorbidities and taking medication regularly. About 75% had sought medical care during the preceding year.	COPD causes a significant impact on the quality of life of caregivers.	Deficit model	34
Simpson et al. (2010)	A day at a time: caregiving on the edge in advanced COPD.	Int J Chron Obstruct Pulmon Dis	Caregiver COPD patients	Qualitative study to better understand the extent and nature of ‘burden’ experienced by informal caregivers in advanced COPD.	Interviews	The analysis of 14 informal caregivers’ interviews yielded the global theme ‘a day at a time,’ reflecting caregivers’ approach to the process of adjusting/coping. Subthemes were: loss of intimate relationship/identity, disease‐related demands, and coping‐related factors.	Findings reflect substantial caregiver vulnerability in terms of an imbalance between burden and coping capacity.	Deficit model	32
Spence et al. (2008)	Active carers: living with chronic obstructive pulmonary disease	Int J Palliative Nurs	Informal caregivers of patients with advanced COPD	Qualitative	Interviews were conducted with seven active family caregivers.	Results confirm that family caregivers provide direct care with little support and assistance.Family caregivers need social and professional support while caring for a patient at home. This would help to ensure that their physical and emotional health does not suffer. There is a need to devise interventions to ensure family caregivers are supported.	Participants reported restricted activities of daily living and some emotional distress. There were knowledge deficiencies among caregivers relating to the COPD illness trajectory and little awareness of the potential of palliative care.	Deficit model/Assets model	32
Weisser et al. (2015)	Experiences of burden, needs, rewards and resilience in family caregivers of people living with Motor Neuron Disease/Amyotrophic Lateral Sclerosis: A secondary thematic analysis of qualitative interviews	Palliative Medicine	Family caregivers of people living with Motor Neurone Disease/Amyotrophic Lateral Sclerosis	Qualitative interviews	24 semi‐structured qualitative interviews conducted longitudinally with 10 family caregivers.	Themes emerged around burden, needs, rewards and resilience.	Resilience included getting active, retaining perspective and living for the moment.Burden, resilience, needs and rewards are interrelated. Caregivers’ ability to cope with caring for a person with Motor Neuron Disease/Amyotrophic Lateral Sclerosis oscillates between positive and negative aspects of caring, being at times active, at times passive.	Assets modelCoping is a non‐linear process, oscillating between different states of mind.	34
Wodskou et al. (2014)	A qualitative study of integrated care from the perspectives of patients with chronic obstructive pulmonary disease and their relatives.	BMC Health Service Research	34 patients with severe or very severe COPD eight of their relatives	Qualitative study	Seven focus groups and five individual interviews two focus groupsData were analysed using inductive content analysis.	Four main categories of experiences of integrated care emerged: 1) a flexible system that provides access to appropriate healthcare and social services and furthers patient involvement; 2) the responsibility of health professionals to both take the initiative and follow‐up; 3) communication and providing information to patients and relatives; 4) coordination and professional cooperation.	The study suggests further studies on impact of caregiving on the informal caregivers, including the impact during and after acute events like COPD‐related hospital admissions	Deficit Model	34

### 3.6 Data abstraction

In keeping with the REA methodology, data were extracted only on results and key data to conduct a quality assessment. For each paper, the following details were extracted: author, year of publication, title, population, design method, results, impact of the disease on caregivers/protective factors, deficit/assets model and scoring for methodological rigour (Blank et al., 2012).

### 3.7 Synthesis

Data were extracted by one reviewer and checked by a second reviewer (Blank et al., 2012).

## 4 Results

### 4.1 Resilience in caregivers in chronic illness

To ensure that their physical and emotional health does not suffer and promote resilience, family caregivers need social and professional support while caring for their chronically ill beloved ones at home (Spence et al., 2008). In caregivers of patient with dementia, social support is described as a moderating factor of resilience. Different types of support seemed to relieve the physical and mental overload caused by stress (Dias et al., 2015).

The imbalance between illness burden and coping capacity in informal caregivers could lead to fatigue and even burnout (Proot et al., 2003). In addition, personal, relational and cultural factors can significantly affect the balance between coping capacity and illness burden, and therefore increase the risk of vulnerability (Simpson et al., 2010). Effective coping with discomfort and nursing needs can help caregivers and lessen the effect of stressors (Papastavrou et al., 2011). Caregivers who learn to accept the new chronic conditions of their beloved one, can redefine the issues related to the illness in a more manageable way and feel they can solve problems more easily (Redinbaugh, Baum, Tarbell, & Arnold, 2003). ‘Family resilience’ is defined as an enduring force that leads a family to change its dynamics of functioning to solve problems associated with stresses encountered. Redinbaugh et al. included this concept in the ‘family resilience model’ by depicting it in the process of change. Outcomes of this process are tranquility and harmony in a family, resulting from a process of transformation, where resilience becomes incorporated as a powerful and propulsive force (Lee et al., 2004).

According to the ‘Assets of Health Model’, positive coping approaches need to be developed by caregivers to continue their caring role. The need to find ways of engaging with caregivers as part of nursing care approach is essential (Hynes, Stokes, & McCarron, 2012). The focus appears to have shifted towards the creation of programs that test an intervention to empower resilience and prevention programs to disseminate resilient attitudes (Earvolino‐Ramirez, 2007). Lin et al. (2013) analysed the concept of resilience among caregivers of children with chronic conditions and declared that ‘the power of resilience enables caregivers to achieve balance, confidence and personal strength’ and that ‘caregivers tended to focus on the positive, and the caregivers’ belief was that a child with a chronic condition is a special favour for them’. Caregiver resilience in the context of caring for chronically ill children can be defined in four main dimensions: disposition patterns, situational patterns, relational patterns and cultural patterns. Some caregivers seem to escape the negative outcomes that taking care of their beloved ones may trigger. Resilience and coping factors may protect caregivers from physical health risks. Therefore, resilient caregivers are proactive in gathering information, developing social support networks, and maintaining balanced relations and collaboration with healthcare professionals (Lin et al., 2013).

Resilience factors in dementia caregiving focus on ‘the broad domains of personal mastery, self‐efficacy and coping style’ (Harmell, Chattillion, Roepke, & Mausbach, 2011). The gradual increase in the levels of personal mastery and self‐efficacy, and the use of positive coping strategies seem to have a protective effect on a variety of health outcomes in dementia caregivers. Caregivers’ self‐efficacy can have a positive impact on life choices, motivation, quality of operation and resistance to adversity (Garcia‐Dia et al., 2013; Harmell et al., 2011). Australian Caregivers of people with stroke who knew how to use active coping strategies reported a stronger relationship with the stroke survivor and a greater appreciation of life. Friends appear as suppliers of valuable support through occasional home visits, practical help and emotional support (El Masry, Mullan, & Hackett, 2013).

Resilience in caregivers of people with Motor Neuron Disease/Amyotrophic Lateral Sclerosis includes getting active, retaining perspective and living for the moment. Burden, resilience, needs and rewards are interrelated. The experience of caregiving oscillates between positive and negative aspects of caring; by being at times active, at times passive (Weisser, Bristowe, & Jackson, 2015).

### 4.2 Resilience as a concept for understanding family caregiving in COPD

COPD patients have some characteristics that are in common with other chronically ill populations in terms of high mortality, re‐hospitalization risk and post‐hospital need for care. COPD is a relatively unpredictable long‐term illness with evident emotional consequences, therefore this type of patient population can indeed be challenging for caregivers (Pinto et al., 2007; Cain & Wicks, 2000).

Physical and functional limitations that COPD patients face on a daily basis, and especially towards the final stages of the disease, require the support of others. COPD patients and their informal caregivers are confronted with increasing limitations because of disease progression, and symptom exacerbations occur more frequently towards the final stages of COPD (Cain & Wicks, 2000; Nakken et al., 2015). COPD also has a profound and pervasive effect on family and friends, and caregivers of patients with chronic disease can experience a high degree of distress (Covinsky et al., 1994; Gabriel et al., 2014). In addition, family members often experience an overwhelming feeling of duty to care for their partners, which combined with a loss of intimacy can lead to psychological distress. (Marques, Jácome, & Cruz, 2015).

In Caress, Luker, and Chalmers's (2010) study, there were numerous examples throughout the data of the impact of the illness on patients and carers. Breathlessness, in particular, was difficult to deal with and resulted in anxiety of future dyspnoeic episodes. Patient's acute exacerbation of COPD can be a potentially traumatic event for caregivers (Simpson et al., 2010). Simpson et al.'s (2010) results reflect substantial caregiver vulnerability in terms of an imbalance between burden and coping capacity, and included comments about the negative impact of the illness on the family, such as the increasing demands of illness on caregivers, and care recipients showing emotional control attitudes. These factors had a negative impact on relationship dynamics and identity. It is therefore important to develop a greater understanding of caregiving psychological distress to help clinicians to identify caregivers who may require more intensive assessment and support mechanisms (Grant et al., 2012). Currently, it is not possible to evaluate the prevalence of psychological distress but current evidence suggests that many factors are related to caregiver psychological distress (Grant et al., 2012).

In Wodskou, Høst, and Godtfredsen (2014) hospital admission for exacerbation was perceived as a difficult experience for several patients with COPD, but felt hospitalization was necessary. In addition, being discharged too soon was described as a factor that could lead to hospital readmission. Wodskou et al. (2014) therefore suggest further studies on the impact of caregiving on informal caregivers, also during and after acute events like COPD‐related hospital admissions.

In Caress et al. (2010) COPD patients’ perspective was to ‘just carry on’ as best they could despite troublesome symptoms and limitations in functioning. The daily life for most patients involved managing day‐to‐day demands linked to the symptoms of the disease. Some patients reported having effective coping strategies to control their anxiety, fear and panic developed through the experience of past COPD symptoms exacerbations. However, for many, coping simply meant limiting those factors that sometimes led to a total cessation of an activity.

The complex interaction between the experience of living with COPD and communication patterns, emotional states, social support and social roles in the family emerged in Gabriel et al. (2014). The results showed the need to develop family‐based interventions for a functional adaptation to COPD that are focused on coping and adapting familiar paths to reduce vulnerability and facilitate a positive adaptation (Gabriel et al., 2014) In Meier, Bodenmann, Mörgeli, and Jenewein (2011), family caregivers provide crucial support to patients with COPD but they experience considerable burdens themselves. The strains of a chronic disease like COPD should also be viewed from a couple's perspective. In pulmonary rehabilitation, the integration of the family member to the rehabilitation process can improve the coping strategies of the family in managing the disease, as well as the couple's sexual functioning and psychological distress (Marques et al., 2015).

## 5 Discussion

The literature review undertaken provides a basis to understand adaptation and resilience in this population. Resilience is potentially a useful concept for understanding family caregiving in adults with COPD. Exploring the interaction between patients and informal caregivers and paying attention to the needs of informal caregivers requires more research (Nakken et al., 2015). Caregiving in COPD patients can be seen as a process, and resilience in COPD caregivers can be described as an enduring ability or capacity that is exhibited as a strength of the caregiver when responding to acute exacerbations, chronic stresses and problem solving for symptoms management (Lin et al., 2013) and which can be further developed (Hicks & Conner, 2013).

The term ‘resilience’ in COPD caregiving is most often understood as using a deficit model of health. Conventional approaches to public health typically seek to identify aspects of burden and stress of the caregiver and fail to take into account the positive effects of caregiving. ‘Taking an asset‐based approach involves mobilizing the skills and knowledge of individuals and the connections and resources in communities and organizations, rather than focusing on problems and deficits’ (NHS Health Scotland, 2011). In Lin et al. (2013) and El Masry et al. (2013) positive aspects of caregiving emerge. This can be read through the theory of the Assets Model of Health and empowering caregivers can enable them to rely less on public services. COPD Caregivers can potentially exhibit several different cognitive and behavioural coping strategies for managing their situation. Caregivers’ ability to cope with caring for a person with COPD – as in other similar chronic conditions – can oscillate between positive and negative aspects of caring (El Masry et al., 2013; Harmell et al., 2011; Lin et al., 2013; Weisser et al., 2015). Caregivers may experience positive caregiving, not only experience distress. In fact, caring for a loved one can be considered as an opportunity for personal growth. Being able to help the patient stay at home as long as possible may be rewarding (Nakken et al., 2015). Family caregiving was found to be rewarding, with caregivers demonstrating a certain amount of resilience, despite the caregiving role entailed significant changes (Spence et al., 2008).

For the professional health care community to support caregivers, resilience must be fully explored in COPD caregivers. Major insight into the role of the home environment is needed to optimize domiciliary management programs (Nakken et al., 2015). A qualitative study may illuminate the complex individual, relational, societal and policy, factors that impact on family caregiving in this group. Current approaches are not enough, and health systems need to actively support caregivers of people with advanced COPD to relieve the burden of caring experienced by caregivers (Hynes et al., 2012; Marques et al., 2015).

A clearer understanding of the concept may support more effective clinical decisions, improve communication between the parties involved, health care professionals, patients and carers, and contribute to improve the planning of nursing care, facilitate clinical research and enhance nursing practice. A better understanding of the physical, emotional, spiritual and relational factors that increase caregivers’ vulnerability can inform new chronic care models better able to support their efforts (Simpson, Young, & Donahue, 2010). Such work may help nurses to understand which supportive nursing interventions are more effective in helping caregivers of adults with COPD.

## 6 Conclusion

The demand for informal caregivers is expected to rise by more than 85% over the next few decades because of the growing population of older adults (Sautter et al., 2014). International policies on long‐term care management recommend several strategies to contrast the negative consequences of providing informal care (Grant et al., 2012). Spouse caregivers in particular need professional support to better manage their daily life as a couple. This appears to the fundamental to ensure higher levels of resilience for the management of COPD. Caring activities should be appropriately spread across patients, partners and health workers. The burden of this responsibility should not fall only on the informal family caregivers (Meier et al., 2011). Professionals should give families a positive feedback from successful experiences of coping, since it strengthens the COPD family resources (Gabriel et al., 2014).

Effective coping with the hardships and demands of caring for family members affected by COPD can support caregivers and reduce the effect of stressors (Papastavrou et al., 2011). Despite the burden, carers can also develop a strong sense of duty to care, who describe the satisfaction they feel as ‘being able to do something useful’ (Spence et al., 2008). Focusing nursing care on a person's health assets, as well as on the positive sides of caring of a relative, together with the traditional approach of the assessment of care needs, can contribute to improved health behaviours and outcomes (Rotegård et al., 2010). If the health systems do not actively engage with caregivers, the current health policy approaches are insufficient to support people with advanced COPD or may even aggravate the burden of care and illness experienced by family carers (Hynes et al., 2012).

This paper has presented an in‐depth analysis of the concept of ‘resilience’ in caregivers, by analysing in particular the possible applications to COPD caregivers. Positive coping strategies need to be developed by caregivers of COPD patients to enable them to continue their caring role. ‘Family resilience is a resource for conquering difficulties, which often manifests in individuals as tranquility, hope and a positive outlook’ (Lee et al., 2004). Therefore, caregivers of COPD adults may experience this when caring for their family members. The model of family coping, that incorporates both family resilience and family functioning, as a property and a process of change, respectively, could apply to caregivers of COPD adults. However, the caregiver population is very heterogeneous and this can make targeted interventions challenging. The stressors and progressive responsibilities that caregivers face on a daily basis, are complex and may consequently require researchers to consider more components, and therapeutic strategies based on evidence to better define the clinical outcomes (Harmell et al., 2011).

## Funding

This research received no specific grant from any funding agency in the public, commercial or not‐for‐profit sectors.

## Conflict of Interest

The authors declare that they have no conflict of interest.

## Author Contributions

FR, SK, GA: Manuscript preparation. FR, SK, AB, LS: Conceptualization of the study.

All authors have agreed on the final version and meet at least one of the following criteria [recommended by the ICMJE (http://www.icmje.org/recommendations/)]:

- substantial contributions to conception and design, acquisition of data or analysis and interpretation of data;
- drafting the article or revising it critically for important intellectual content.
